# Characterization and Quantification of Luteolin-Metal Complexes in Aqueous Extract of Lonicerae Japonicae Flos and Huangshan Wild Chrysanthemum

**DOI:** 10.1155/2021/6677437

**Published:** 2021-03-11

**Authors:** Weilan Cai, Yunhao Xiong, Manman Han, Zhimin Li, Liang Peng, Hongyi Zhang, Qin Zou, Lin Wu, Qingling Ye, Linfeng Liao

**Affiliations:** Jiangxi Provincial Key Laboratory of Drug Design and Evaluation, School of Pharmacy, Jiangxi Science & Technology Normal University, Nangchang, Jiangxi 330013, China

## Abstract

Luteolin is a flavonoid compound widely found in vegetables, fruits, and medicinal plants. In this study, the reaction conditions for luteolin and five metal ions (Ca^2+^, Mg^2+^, Zn^2+^, Fe^3+^, and Cu^2+^) to form complexes in hot water were optimized, which was at a molar ratio of 1 : 1 for luteolin and metal ions at 90°C in a volume of 20 mL for 2 h, and the ability of luteolin to form complexes with Cu^2+^ was the strongest. The DPPH scavenging test showed that luteolin exerted a dose-dependent effect on the clearance of free radicals; luteolin-Cu^2+^ complexes and luteolin-Fe^3+^ complexes accentuated the clearance of free radicals. Furthermore, we used high performance liquid chromatography (HPLC) to analyze luteolin in samples from two medicinal plants, obtained from the dissolution of aqueous extracts in two different solvents. The results showed that the peak areas for luteolin in the samples dissolved in 20% formic acid-methanol were significantly larger than those from the samples dissolved in methanol alone, with increases in the peak area being 135.6% (Lonicerae Japonicae Flos), and 161.16% (Huangshan wild chrysanthemum). The aforementioned results indicate that complexes formed from organic compounds and metal ions are present in the decoction of a plant.

## 1. Introduction

Plants are an important source of many biologically active and clinically relevant organic compounds, including phenylpropanoid, flavonoid, anthraquinone, steroids, terpenoids, and alkaloids [1]. They also contain many metal ions such as calcium, magnesium, iron, and copper that form complexes with organic compounds which perform multiple biological activities [2]. Phenolic hydroxyl, carbonyl, carboxyl, sulfhydryl, and amino groups and nitrogen-containing aromatic rings are nearly universally present in organic compounds and may readily form complexes with metals. Rybak and Ruzik [3] found that manganese formed complexes with rutin, alizarin, and asperulosidic acid in noni juice. Using *µ*HPLC-ESI–MS/MS, Wojcieszek et al. [4] detected seven types of copper complexes and four types of zinc complexes in ionic liquid and pectinase extracts of *Lycium barbarum*. Weber and Konieczyński [5] detected manganese and magnesium complexes in Folium Betulae (birch leaves), Folium Menthae (peppermint leaves), and Radix Taraxaci (dandelion roots) by size-exclusion chromatography combined with atomic absorption spectroscopy. Using the membrane filter and macroporous resin, Liu et al. [6] identified by flame atomic absorption spectrometry many complexes between zinc and proteins or polycarbohydrates in the decoction of *Flammulina velutipes.* These studies indicated that multiple complexes of ions and organic compounds are present in plants in nature.

Luteolin is a flavonoid that is widely present in nature and is mainly distributed in medicinal herbs such as Lonicerae Japonica Flos, *Origanum vulgare*, chrysanthemum, and rosemary and in foods including peanuts, carrots, celery, cucumber, and buckwheat. [7, 8] Luteolin possesses many biological activities including antioxidant [9, 10], antibacterial [11], antiviral [12], and antitumor activities [13, 14] and also confers cardiocerebrovascular protection [15, 16]. The phenolic hydroxyl and carbonyl groups have intact *π* conjugation and strong superdelocalizability and proper planar structure, which facilitate complex formation between luteolin and ions.

In the current study, we investigated luteolin ion complex formation by measuring postreaction luteolin content by high performance liquid chromatography (HPLC). We further determined the effects of reaction temperature, volume, and time on the formation of complexes between luteolin and five types of ion including calcium, magnesium, iron, zinc, and copper in the aqueous extract. We also studied the effects of ions on free radical scavenging activities of luteolin. Finally, we determined the presence of luteolin ion complexes in Lonicerae Japonica Flos (*L. japonica*) and Huangshan wild chrysanthemum by HPLC analysis of aqueous extract of *L. japonica* and Huangshan wild chrysanthemum in methanol and 20% formic acid-methanol.

## 2. Materials and Methods

### 2.1. Chemicals

Luteolin was purchased from Haochen Biotechnology Co. (Shaanxi, China), and its purity was confirmed to be greater than 98% by HPLC. Luteolin standard was purchased from the National Institute for the Control of Pharmaceutical and Biological Products (Beijing, China). Methanol (HPLC grade and mass spectrometry grade) was purchased from Merker, Germany; formic acid (HPLC grade) and deuterated methanol were purchased from Aladdin, USA, and 1,1-dipheny-2-trinitrophenylhydrazine (DPPH) was purchased from TCI Chemicals (Shanghai, China). Magnesium chloride hexahydrate, anhydrous calcium chloride, anhydrous zinc chloride, iron chloride hexahydrate, copper chloride dihydrate, ethyl acetate, and DMSO were all analytical grade. Weight of chemicals used was measured using an XS203S electronic scale (METTLER TOLEDO, Swiss).

### 2.2. Plants

*L. japonica* and Huangshan wild chrysanthemum were purchased from Yifeng Pharmacy (Nanchang, China). *L. japonica* was grown in Henan Province, China, and Huangshan wild chrysanthemum was grown in Anhui Province, China. They were officially authenticated by the School of Pharmacy, Jiangxi Science and Technology Normal University. *L. japonica* was dried flower buds of *Lonicera japonica* Thunb., and Huangshan wild chrysanthemum was the dried capitulum of *Dendranthema morifolium* (Ramat) Tzvel. cv. Gongju.

### 2.3. Preparation of Luteolin Standard Curve

HPLC separations were performed on YMC-Pack ODS-A C18 chromatographic column (4.6 mm × 150 mm, 5 *µ*m) with 30°C column temperature. Luteolin was eluted using 2 mmol L^−1^ ammonium formate solution (0.1% formic acid) (A) and methanol (B) (60 : 40, V/V) as the mobile phase with 60% B isocratic elution. The flow rate was 1.0 mL·min^−1^. The injection volume was 1 *μ*L. The wavelength of the VWD detector was set at 350 nm.

Luteolin methanol stock solution (2.5 mmol/L) was prepared and diluted into the following concentrations: 2.25, 2.0, 1.75, 1.5, 1.25, 1.0, 0.75, 0.5, and 0.25 mmol/L. The peak area of HPLC was determined. Luteolin standard curve was plotted with the HPLC peak area as the *x*-axis and luteolin concentrations as the *y*-axis. The following regression equation was derived:

(1) $$\begin{array}{c} Y=0.001X+0.0008\left(R²=0.9999\right), \end{array}$$

indicating that luteolin concentration from 0.25 mmol/L to 2.5 mmol/L had a good linear relationship.

### 2.4. ^1^H-NMR

Luteolin (0.1 mmol) was allowed to react with 0.02 mmol anhydrous calcium chloride, anhydrous zinc chloride, magnesium chloride hexahydrate, iron chloride hexahydrate, or copper chloride dihydrate in 40 mL distilled water at 90°C for 4 h under heat backflow using a Hei-VAP rotary evaporator (Heidolph, Germany) and mixed with a DF-101S heat collection type isothermal magnetic heating stirrer (Yuhua Co., Gongyi, Henan, China). Thereafter, the reactants were spun dry and washed with distilled water several times to remove ions that did not form complexes with luteolin. The remaining solid portion was washed several times with 100 mL ethyl acetate, and after the solid portion was spun dry, it was dissolved in deuterated methanol. Free luteolin and the reactants in deuterated methanol were then studied by nuclear magnetic resonance (NMR) spectroscopy as routinely performed using a Bruker AVANCE 400-MHz spectrometer.

### 2.5. Determination of Reaction Conditions

To determine the effect of reaction temperature on luteolin ion salt complex formation, we mixed 0.1 mmol luteolin with 0.02 mmol anhydrous calcium chloride, anhydrous zinc chloride, magnesium chloride hexahydrate, iron chloride hexahydrate, or copper chloride dihydrate in 40 mL distilled water. The reaction was allowed to proceed for 4 h under heat backflow at a temperature of 30°C, 50°C, 70°C, or 90°C. Furthermore, to determine the effect of reaction time on luteolin ion salt complex formation, we carried out the above reaction at 90°C for 0.5, 1, 2, and 4 h, respectively. In addition, to determine the effect of reaction volume on luteolin ion salt complex formation, we carried out the above reaction in 20, 40, 80, or 160 mL distilled water at 90°C for 2 h. We also investigated the effect of molar ratios of luteolin to ion salts on luteolin ion salt complex formation at a ratio of 10 : 1, 5 : 1, 2 : 1, and 1 : 1 (0.1 mmol luteolin added to 0.002, 0.004, 0.01, and 0.02 mmol anhydrous calcium chloride, anhydrous zinc chloride, magnesium chloride hexahydrate, iron chloride hexahydrate, or copper chloride dihydrate, respectively). The reaction was allowed to proceed for 2 h under heat backflow at 90°C. Finally, we examined the ability of luteolin to complex with different ion salts. Luteolin (0.1 mmol) was allowed to react with 0.1 mmol anhydrous calcium chloride, anhydrous zinc chloride, magnesium chloride hexahydrate, iron chloride hexahydrate, or copper chloride dihydrate in 20 mL distilled water at 90°C under heat backflow for 2 h. Free luteolin without ion salt served as blank control.

The reactants were spun dry and washed with distilled water several times to remove ions that did not form complexes with luteolin. The remaining solid portion was washed several times with 100 mL ethyl acetate, and after the ethyl acetate lotion was spun dry, it was dissolved in 20 mL methanol. Luteolin content was analyzed by HPLC as described above.

### 2.6. DPPH Scavenging Test

Luteolin (0.1 mmol) was allotted to react with 0.1 mmol anhydrous calcium chloride, magnesium chloride hexahydrate, iron chloride hexahydrate, or copper chloride dihydrate in 20 mL distilled water at 90°C under heat backflow for 2 h. The reactants were spun dry and dissolved in DMSO in 10 mmol/L stock solution (using the molar concentration of luteolin). Luteolin and the 4 ion salts were also prepared in equivalent molar concentration. Radical scavenging capacity was evaluated by a DPPH radical test as previously described [17]. The experiments were performed three times independently in triplicate, and the mean value was used to calculate clearance using the following equation:

(2) $$\begin{array}{c} \text{Clearance}\left(\%\right)=\left(1-\frac{A_{s}-A_{c}}{A_{0}}\right)\times 100\%, \end{array}$$

where As represents the mean absorbance of the sample and DPPH, Ac represents the mean absorbance value of the sample, and *A*_0_ represents the mean absorbance value of DPPH solution.

### 2.7. HPLC Analysis

*L. japonica* and Huangshan wild chrysanthemum (20 g each) were dissolved in 200 mL distilled water and after soaking at room temperature for 30 min, were subjected to heat backflow decoction at 95°Cfor 2 h. After filtration, the filtrate was spin-dried to obtain dried decoction. The dried decoction was dissolved in 20 mL methanol or 20 mL 20% formic acid-methanol, and after sonication for 1 min, 1 mL solute was filtered using 0.22 *µ*m membrane and then subjected to HPLC.

HPLC separations were performed on an Agilent Eclipse XDB-C18 analytical column (250 mm × 4.6 mm, 5 *μ*m) with 30°C column temperature. Samples were eluted using 2 mmol L^−1^ ammonium formate solution (0.2% formic acid or methanol) (A) and acetonitrile (B) (60 : 40, V/V) as the mobile phase 0–80 min, 20% B ⟶ 60% B; 80–100 min, 60% B ⟶ 100% B. The flow rate was 0.2 mL·min^−1^. The injection amount was 10 *μ*L. The wavelength of the DAD detector was set at 350 nm.

## 3. Results

### 3.1. Effect of Reaction Temperature on Luteolin-Metal Complex Formation

**Figure 1:**
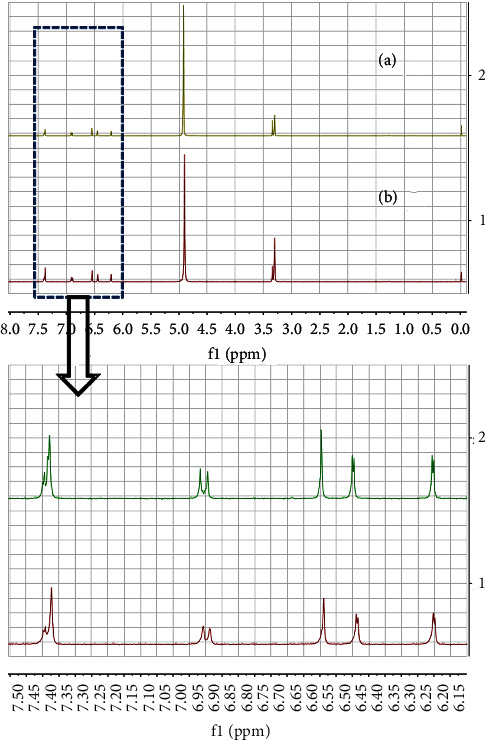
Luteolin was allowed to react with anhydrous calcium chloride, anhydrous zinc chloride, magnesium chloride hexahydrate, iron chloride hexahydrate, or copper chloride dihydrate. After removal of free ion salts, the reactants were analyzed by ^1^H-NMR spectroscopy. (a) Free luteolin was used as a control; (b) ethyl acetate solute.

^1^H-NMR spectroscopy showed that after its reaction with anhydrous calcium chloride, magnesium chloride hexahydrate, iron chloride hexahydrate, or copper chloride dihydrate, luteolin complexes in ethyl acetate exhibited similar spectra to those of free luteolin (Figure 1), indicating that luteolin did not completely react with the five types of ion salts, and luteolin ion salt complexes were not dissolvable in ethyl acetate. Therefore, in subsequent experiments, HPLC was used to quantify luteolin content in ethyl acetate, with higher luteolin content in ethyl acetate indicating a lower capacity to form complexes with these ions.

**Figure 2:**
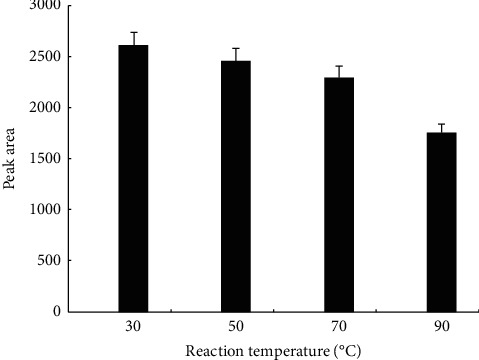
Effect of reaction temperature on luteolin-metal complex formation. Luteolin (0.1 mmol) was mixed with 0.02 mmol anhydrous calcium chloride, anhydrous zinc chloride, magnesium chloride hexahydrate, iron chloride hexahydrate, or copper chloride dihydrate in 40 mL distilled water. The reaction was allowed to proceed for 4 h under heat backflow at a temperature of 30°C, 50°C, 70°C, or 90°C. After free ion salts were removed, the reactants were analyzed by HPLC as described in methods. The experiment was performed at least three times independently.

We then allowed luteolin to react with anhydrous calcium chloride, magnesium chloride hexahydrate, iron chloride hexahydrate, or copper chloride dihydrate at different temperatures to investigate the effect of reaction temperature on luteolin ion complex formation. We observed a gradual reduction in the peak area of luteolin as the reaction temperature rose from 30°C to 90°C (Figure 2). At 90°C, the peak area of luteolin was 67.2% of that at 30°C. The reaction temperature was set at 90°C for subsequent experiments.

### 3.2. Effect of Reaction Time and Volume on Luteolin-Metal Complex Formation

**Figure 3:**
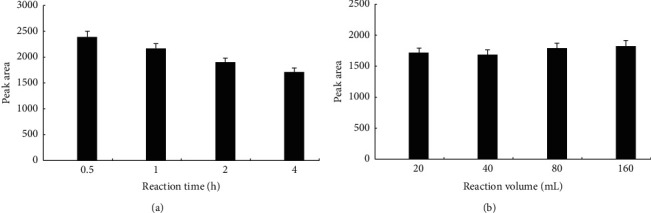
Effects of reaction time and volume on luteolin-metal complex formation. (a) Luteolin (0.1 mmol) mixed with 0.02 mmol anhydrous calcium chloride, anhydrous zinc chloride, magnesium chloride hexahydrate, iron chloride hexahydrate, or copper chloride dihydrate was allowed to proceed for 0.5, 1, 2, or 4 h under heat backflow at a temperature of 90°C. (b) The reaction in (a) was carried out in 20, 40, 80, or 160 mL distilled water at 90 for 2 h. After free ion salts were removed, the reactants were analyzed by HPLC as described in methods. The experiment was performed at least three times independently in triplicate.

We also delineated the effect of reaction time on luteolin ion complex formation. The amount of luteolin gradually decreased as the reaction time increased (Figure 3(a)). At 4 h, the peak area of luteolin decreased by 9.7% versus that at 2 h, suggesting that most luteolin ion complexes had already formed by 2 h. The reaction time was set at 2 h for subsequent experiments. We further studied the effect of solvent volume on luteolin ion complex formation. We observed no apparent difference in the content of luteolin in ethyl acetate among different reaction volumes (Figure 3(b)). The peak area at a volume of 40 mL was the smallest and declined only by 1.98% versus that at a volume of 20 mL, which fell within the standard error of weighing. The peak area at a volume of 80 mL and 160 mL increased only by 6.00% and 9.04%, respectively, compared with that at a volume of 20 mL. This could be due to lower ion concentrations with increased volume, which does not facilitate complexation. The reaction volume was set at 20 mL for subsequent experiments.

### 3.3. Effect of Molar Ratios of Luteolin to Ions and Different Types of Ions on Luteolin Ion Complex Formation

**Figure 4:**
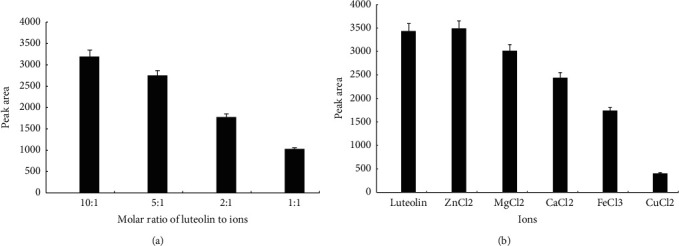
(a) Effect of the molar ratio of luteolin to metal ions on the formation of luteolin-metal complex (*n* = 3). (b) Effect of different types of metal ions on luteolin-metal complex formation (*n* = 3).

Luteolin was added at a molar ratio of 10 : 1, 5 : 1, 2 : 1, or 1 : 1 to ions. As shown in Figure 4(a), when the molar ratio of luteolin to ions was 1 : 1, the amount of luteolin in ethyl acetate was only 31.8% of the amount of luteolin when the luteolin to ion ratio was 10 : 1, suggesting that the amount of ions had a marked effect on luteolin ion complex formation: the greater the amount of ions was, the greater the amount of luteolin ion complexes was formed. In subsequent studies, the ratio of luteolin to ions was maintained at 1 : 1.

We further examined luteolin ion complex formation between luteolin and different types of ions. Under condition for water decoction at 90°C under heat backflow for 2 h, the peak area was the smallest following luteolin and Cu^2+^ reaction (Figure 4(b)). The peak area after luteolin and Zn^2+^ reaction increased 1.82% compared to that with no ions, indicating virtually no formation of the luteolin-Zn^2+^ complex. The ability to form luteolin ion complexes was as follows: Cu^2+^ > Fe^3+^ > Ca^2+^ > Mg^2+^ > Zn^2+^.

### 3.4. Effect of Metal Ions on Clearance of Free Radicals by Luteolin

Luteolin exhibited a dose-dependent effect on clearance of free radicals. The clearance rate of free radicals was 18.34% for luteolin at 0.10 mmol, which shot up to 80.27% for luteolin at 1.0 mmol (Table 1). The clearance rate of free radicals stood at 89.69%, 73.44%, 94.63%, and 79.46% for luteolin-Fe^3+^ complexes, luteolin-Mg^2+^ complexes, luteolin-Cu^2+^ complexes, and luteolin-Ca^2+^ complexes at 1.0 mmol/L, respectively. In the absence of luteolin, the clearance rate of free radicals was 29.81%, 7.0%, 39.39%, and 5.70% for Fe^3+^, Mg^2+^, Cu^2+^, and Ca^2+^ salt only at 1.0 mmol/L, respectively. These findings suggested that luteolin-Cu^2+^ complexes and luteolin-Fe^3+^ complexes accentuated the clearance of free radicals.

### 3.5. Luteolin-Metal Complexes in *L. japonica* and Huangshan Wild Chrysanthemum Decoction

**Figure 5:**
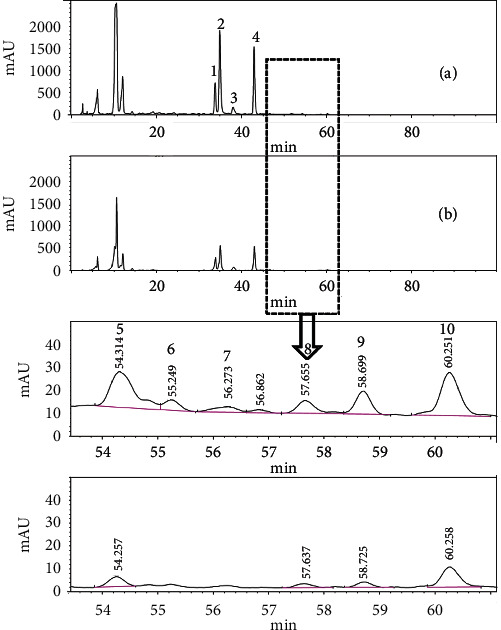
The chromatographic profiles of the 20% formic acid-methanol extract (a) and methanol extract (b) of *L. japonica* decoction.

**Figure 6:**
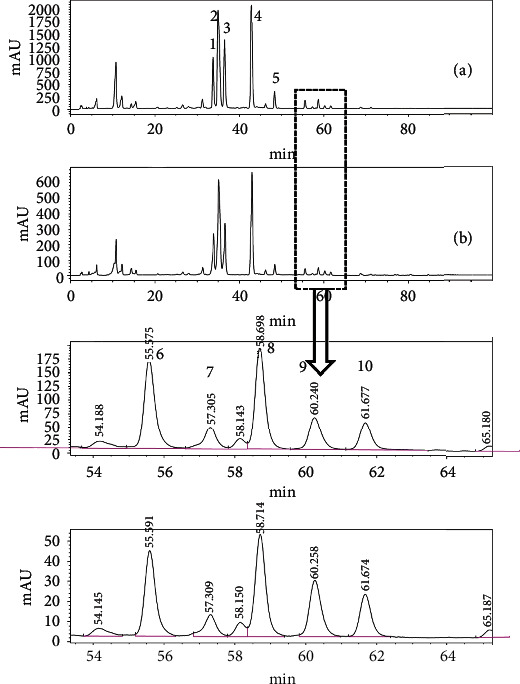
The chromatographic profiles of the 20% formic acid-methanol extract (a) and methanol extract (b) of Huangshan wild chrysanthemum decoction.

The chromatographic profiles of the methanol extract and 20% formic acid-methanol extract of *L. japonica* decoction are shown in Figure 5, and the retention time and peak area of ten well-isolated chromatographic peaks are given Table 2. The chromatographic profiles of the methanol extract and 20% formic acid-methanol extract of Huangshan wild chrysanthemum decoction are shown in Figure 6, and the retention time and peak area of ten well-isolated chromatographic peaks are given Table 3.

The retention time of the methanol extract of *L. japonica* decoction was 60.26 ± 0.02 min, and the peak area was 1091.53 ± 13.15. The retention time of the 20% formic acid-methanol extract of *L. japonica* decoction was 60.24 ± 0.02 min, and the peak area was 1094.37 ± 9.28. Luteolin contains multiple phenolic hydroxyl groups and is weakly acidic, which, to a certain extent, modifies its chromatographic behavior; therefore, in the 20% formic acid-methanol extract, the peak appeared earlier than that in the methanol extract. However, there was no difference in the peak area between the two extracts.

The chromatographic profiles of the methanol extract and 20% formic acid-methanol extract of *L. japonica* decoction and Huangshan wild chrysanthemum decoction were largely in agreement with one another (Figures 5 and 6). In Huangshan wild chrysanthemum decoction, the number of chromatographic peaks, resolution, and peak form were identical in the two extracts. *L. japonica* decoction had two apparent chromatographic peaks at 55.249 min and 56.273 min in the 20% formic acid-methanol sample. Though no apparent chromatographic peaks were observed around the above two retention times using automatic detection software, two small protuberances, which were similar in shape to the two chromatographic peaks in the 20% formic acid-methanol sample, were present in the baseline by manual examination, suggesting the presence of similar compounds at 55.249 min and 56.273 min.

We noticed that luteolin-metal complexes were slightly dissolved in methanol but completely dissolved in 20% formic acid-methanol into ligands and corresponding metal ions (data not shown). The chromatographic peak area of both *L. japonica* and Huangshan wild chrysanthemum decoction in 20% formic acid-methanol samples was higher than that in the methanol samples (Tables 2 and 3). The chromatographic peak of luteolin in *L. japonica* decoction in 20% formic acid-methanol samples increased 1.36 fold, while that of Huangshan wild chrysanthemum decoction increased 1.61.

## 4. Discussion

Luteolin is one of the important plant metabolites. Due to the presence of carbonyl and hydroxyl groups, luteolin can coordinate metal ions to form complexes, and the metal complexes have shown higher biological effects than free luteolin. Over recent years, many luteolin-metal ion complexes have been synthesized including luteolin complexes with copper [18, 19], cadmium [20], chromium [20, 21], vanadium [22–24], aluminum [24–26], manganese [27], zinc [28, 29], iron (III) [30, 31], and rare earth elements [32]. However, these luteolin-metal ion complexes are synthesized in an organic solvent or alkaline aqueous solution. Anna et al. demonstrated by NMR that the energetically favored Zn chelation sites of the 1 : 1 zinc-luteolin complexes were 4 = O and 5-OH [25]. Rygula et al. [26] showed that, after dissolving in sodium hydroxide, luteolin and Zn^2+^ mainly coordinate at 3′-OH and 4′-OH of luteolin in Tris-HCl (pH 7.4). Hao et al. [27] detected three reaction products, such as [(Lu-2H)^2−^ + Fe^3+^+NO_3_^−^ + H^+^], [(Lu-2H)^2−^ + Fe^3+^+2CH_3_CH_2_OH], and [(Lu-2H)^2−^ + Fe^3+^+NO_3_^−^+2CH_3_CH_2_ −OH + *H*^+^], by ESI-TOF-MS when luteolin reacted with Fe (NO_3_)_3_·9H_2_O in ethanol; moreover, these reaction products enhanced the clearance of DPPH free radicals. As most edible and medicinal plants are decocted in water before use, the current study used distilled water as solvent to investigate the formation of complexes between luteolin and metal ions in plant aqueous extracts. Our study demonstrated that luteolin and Zn^2+^ did not form complexes in distilled water, while the reaction products of luteolin and Fe^3+^ lowered the clearance of DPPH free radicals by luteolin, suggesting that reaction solvent affects the ability of luteolin to complex with metal ions, chelation sites, and activities of complexes.

In analyzing methanol and 20% formic acid-methanol samples of *L. japonica* and Huangshan wild chrysanthemum decoction, we took great efforts to minimize variations in weighing and extracting the medicinal herbs and divided a decoction into two parts equivalent in volume which was then spun dry and dissolved in solutions. Furthermore, filtration was performed at 95°C in the waterbath to prevent precipitation of compounds due to decrease in filtrate temperature, leading to great variations in results. HPLC revealed that the area of chromatographic peaks for luteolin and other compounds increased to various extents in *L. japonica* and Huangshan wild chrysanthemum decoction in 20% formic acid-methanol samples. Though L. *japonica* and Huangshan wild chrysanthemum contain, apart from luteolin, glycosides from luteolin complexation with carbohydrates, we used moderate to strong acid, formic acid, which was of relatively low concentration in the solution, and the dissolution condition was relatively mild; consequently, the luteolin peak area in 20% formic acid-methanol solution markedly increased. Increased luteolin content was unlikely caused by increased luteolin-glycoside complexes but more likely due to dissolution of luteolin-metal complexes in the presence of formic acid. Moreover, the amplitude of increase in the luteolin peak area of the decoctions of the two plants was different in 20% formic acid-methanol, which may be due to the difference in the types and contents of metal ions in the two plants.

## 5. Conclusion

The current study has demonstrated that, apart from organic compounds and metal ions, complexes formed from organic compounds and metal ions are also present in the decoction of a plant. These findings suggest that investigators should also characterize and study the biological significance of organic compound-metal ion complexes in plants.

## Figures and Tables

**Table 1:** Effect of luteolin, metal salt and luteolin, and metal ion reaction products on clearance of DPPH free radicals.

Concentration, mmol/L	Clearance%								
	Luteolin	Fe complex	Mg complex	Cu complex	Ca complex	Fe salt	Mg salt	Cu salt	Ca salt
1.0	80.27	89.69	73.44	94.63	79.46	29.81	7.00	39.39	5.70
0.1	69.66	72.66	73.30	86.54	76.02	16.9	−0.84	7.83	4.79
0.01	18.34	15.03	31.21	36.72	34.65	7.7	−1.29	0.08	4.26

**Table 2:** Retention time and peak area of *L. japonica* decoction ($\bar{X}\pm S$, *n* = 3).

No.	20% formic acid-methanol sample		Methanol sample		Increase in peak area (%)
	Retention time	Peak area	Retention time	Peak area	
1	33.806	18562.99 ± 1078.91	33.898	8072.82 ± 487.77	129.94
2	34.925	55410.47 ± 3220.53	35.055	16619.99 ± 1004.20	233.40
3	38.134	8282.763 ± 481.41	38.186	2587.756 ± 156.35	220.08
4	42.941	37570.57 ± 2183.65	42.982	13548.2 ± 818.59	177.31
5	54.314	486.37 ± 28.27	54.257	88.5658 ± 5.35	449.17
6	55.249	91.60 ± 5.32	Not detected	Not detected	—
7	56.273	73.73 ± 4.29	Not detected	Not detected	—
8	57.655	141.49 ± 8.22	57.637	33.18 ± 2.00	326.48
9	58.699	195.47 ± 11.36	58.725	43.25 ± 2.61	351.95
10	60.251 (luteolin)	484.24 ± 28.14	60.258 (luteolin)	205.54 ± 12.42	135.60

**Table 3:** Retention time and peak area of Huangshan wild chrysanthemum decoction ($\bar{X}\pm S$, *n* = 3).

No.	20% formic acid-methanol sample		Methanol sample		Increase in peak area (%)
	Retention time	Peak area	Retention time	Peak area	
1	33.77	25286.71 ± 1699.06	33.908	7805.70 ± 689.08	223.95
2	34.937	72512.27 ± 4872.24	35.089	24210.14 ± 2137.26	199.51
3	36.446	34578.05 ± 2323.36	36.556	9695.21 ± 855.89	256.65
4	42.804	60551.63 ± 4068.58	42.970	19756.52 ± 744.10	206.49
5	48.329	7949.30 ± 534.13	48.391	1785.63 ± 157.63	345.18
6	55.575	3824.70 ± 256.99	55.591	936.94 ± 82.71	308.21
7	57.305	1077.09 ± 72.37	57.309	224.54 ± 19.82	379.69
8	58.698	3877.06 ± 260.51	58.714	1091.58 ± 96.36	255.18
9	60.240 (luteolin)	1538.59 ± 103.38	60.258 (luteolin)	664.29 ± 58.64	131.61
10	61.677	1253.40 ± 84.22	61.674	479.94 ± 42.37	161.16

## Data Availability

The datasets used and/or analyzed during the current study are available from the corresponding author upon request.
